# In Silico Study on Tumor-Size-Dependent Thermal Profiles inside an Anthropomorphic Female Breast Phantom Subjected to Multi-Dipole Antenna Array

**DOI:** 10.3390/ijms21228597

**Published:** 2020-11-14

**Authors:** Piotr Gas, Arkadiusz Miaskowski, Mahendran Subramanian

**Affiliations:** 1 Department of Electrical and Power Engineering, Faculty of Electrical Engineering, Automatics, Computer Science and Biomedical Engineering, AGH, University of Science and Technology, Mickiewicza 30 Avenue, 30-059 Krakow, Poland; 2 Department of Applied Mathematics and Computer Sciences, Faculty of Production Engineering, University of Life Sciences in Lublin, Akademicka 13 Street, 20-950 Lublin, Poland; arek.miaskowski@up.lublin.pl; 3 Department of Bioengineering and Department of Computing, Royal School of Mines, Imperial College London, London SW7 2AZ, UK; m.subramanian@imperial.ac.uk; 4 Faraday-Fleming Laboratory, London, W14 8TL, UK

**Keywords:** regional deep hyperthermia planning, breast cancer, dipole antenna array, multi-objective optimization, specific absorption rate, numerical modelling, FDTD method

## Abstract

Electromagnetic hyperthermia as a potent adjuvant for conventional cancer therapies can be considered valuable in modern oncology, as its task is to thermally destroy cancer cells exposed to high-frequency electromagnetic fields. Hyperthermia treatment planning based on computer in silico simulations has the potential to improve the localized heating of breast tissues through the use of the phased-array dipole applicators. Herein, we intended to improve our understanding of temperature estimation in an anatomically accurate female breast phantom embedded with a tumor, particularly when it is exposed to an eight-element dipole antenna matrix surrounding the breast tissues. The Maxwell equations coupled with the modified Pennes’ bioheat equation was solved in the modelled breast tissues using the finite-difference time-domain (FDTD) engine. The microwave (MW) applicators around the object were modelled with shortened half-wavelength dipole antennas operating at the same 1 GHz frequency, but with different input power and phases for the dipole sources. The total input power of an eight-dipole antenna matrix was set at 8 W so that the temperature in the breast tumor did not exceed 42 °C. Finding the optimal setting for each dipole antenna from the matrix was our primary objective. Such a procedure should form the basis of any successful hyperthermia treatment planning. We applied the algorithm of multi for multi-objective optimization for the power and phases for the dipole sources in terms of maximizing the specific absorption rate (SAR) parameter inside the breast tumor while minimizing this parameter in the healthy tissues. Electro-thermal simulations were performed for tumors of different radii to confirm the reliable operation of the given optimization procedure. In the next step, thermal profiles for tumors of various sizes were calculated for the optimal parameters of dipole sources. The computed results showed that larger tumors heated better than smaller tumors; however, the procedure worked well regardless of the tumor size. This verifies the effectiveness of the applied optimization method, regardless of the various stages of breast tumor development.

## 1. Introduction

Neoplastic diseases and the growing number of deaths caused by them is a challenging major global health issue [1]. Long-term cancer treatment negatively affects the psyche of patients, significantly decreases their social and economic wellbeing, and deteriorates their quality of life. Breast carcinoma has been recognized and diagnosed since ancient times [2]. However, modern medicine enables novel and innovative female breast-conserving therapies, including hyperthermia, to cure malignant tumors [3,4]. Hyperthermia is a specialized oncological therapy aimed at the permanent destruction of cancer cells or a significant inhibition of their multiplication at high temperatures in the range of 40–46 °C [5] or higher through the use of thermal ablation treatment [6,7]. In clinical practice, hyperthermia is an important adjunct treatment that is combined with other anti-cancer modalities such as radiotherapy, chemotherapy, or immunotherapy. Many recent studies show that combination therapy significantly increases the effective treatment of densely vascularized and low-oxygenated carcinomas [8,9].

In electromagnetic (EM) hyperthermia, the heating of pathological tissues is accomplished by depositing radiofrequency (RF) or microwave (MW) energy into a region of interest (ROI). The main disadvantages of different ablation techniques, including radiofrequency ablation [10], microwave ablation [11,12], laser ablation [13], high-intensity focused ultrasound ablation [14], and cryoablation [15], are both the invasiveness of the treatments and their limited access to deep-seated tumors, e.g., in the case of lung, liver, brain, prostate, and cervical cancers [16]. To achieve a localized EM-energy that focuses on deep-rooted tumors without affecting neighboring normal tissues, other techniques are required. In the treatment of tumors that are localized a few or several centimeters below the surface of the skin, phased array antennas around the patient’s body can be used [17]. Adjusting the EM energy of the antennas’ matrix and achieving the most effective heat dose for tumor damage is an extremely challenging task. Phased arrays often utilize optimization procedures in line with the specific absorption rate (SAR) parameter, whereby the amplitude, phase, or frequency of the excitation signal produced by monopole antennas [18], dipole antennas [19,20], patch antennas [21], or more complex applicators [22] can be adjusted. They operate in broadband (treatment of surface tumors) [16] or narrowband regimes (treatment of deep-seated tumors) [23] at one or more frequencies [18]. An example of the medical usage of phased array antennas would be the BSD-2000 hyperthermia system including Sigma-Eye applicators produced by the BSD Medical Corporation [24]. The matrices of various antennas and coils are commonly employed in treating other disorders [25,26]. It is also possible to use interstitial applicators, inserted into the cancer tissue, but their percutaneous application is not always possible for medical reasons. Noninvasive techniques appear to be safer for patients but require the appropriate targeting of EM energy to limit harmful heating of normal tissues surrounding the tumor. In order to eliminate the ”hot spots” and separate them from the treated tissue, boluses with cold water or hydrogels placed between the applicator and the tissue might be employed [27]. The concentration of EM energy in the cancerous area can also be obtained after placing magnetic nanoparticles (MNPs) within the tumor; however, in such cases, due to the safety procedures, much lower frequencies should be applied [28,29,30,31,32,33]. Importantly, various minimally invasive techniques for hyperthermic or ablative heating of tumors require continuous intensive research, with a concentrated effort by scientists to improve their effectiveness in uniform heating of the ROI and reducing possible side effects [34].

This paper gives the theoretical background and describes the multi-objective optimization procedure for regional deep hyperthermia planning using a multi-element dipole antenna array based on the finite-difference time-domain (FDTD) simulation. The described problem investigates the use of the linearity and superposition of dipole antenna signals. The selection of optimal dipole sources properties seems to be a non-trivial issue. The current paper omits the technical aspects of supplying individual dipole antennas, focusing only on the optimization of their basic parameters, in terms of the phase and input power, in order to achieve optimal treatment of breast tumors through the thermal effects of MW radiation. The multi-objective optimization algorithm, also used in [17,35], adjusts the phase and powers of a dipole antenna array for a specific clinical case using cancer-targeted EM power deposition in the terms of the SAR parameter. This professional tool that uses the computational capabilities of the commercially available software Sim4Life [36], is discussed in an example of female breast cancer. Finally, we investigate how tumor temperature, derived from optimal antenna array settings, depends on the size of the tumor. Only detailed numerical tests on female breast tumor models of various sizes will give the readers a real insight into how the optimization of crucial parameters in the dipoles can influence heating of the targeted tumor. Such a practice verifies the effectiveness of the applied optimization method, regardless of the various stages of breast tumor development. In this paper, the authors’ contribution to the current state of knowledge about MW hyperthermia is the application of the aforementioned optimization procedure using an anatomically correct female breast model, which was combined with the Pennes’ bioheat equation and non-linear perfusion in the tumor together with its various sizes. Such an investigation has, to the best of the authors’ knowledge, never been published before.

## 2. Female Breast Carcinoma

### 2.1. Statistics, Anatomy, and Treatment

**Figure 1:**
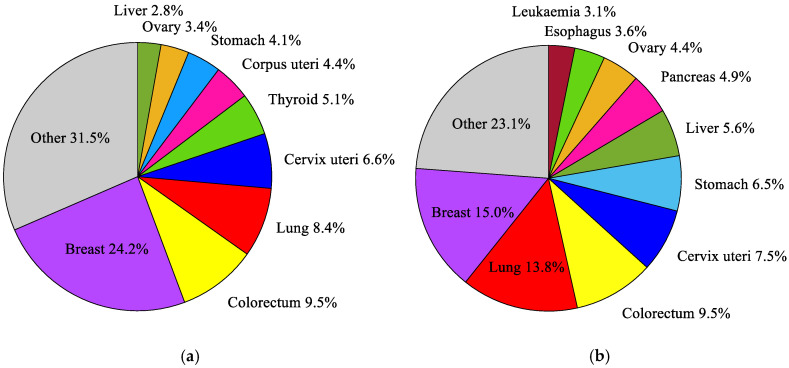
The most common cancers occurring in the world’s female population in 2018: (**a**) incidence; (**b**) mortality, according to the global cancer database GLOBOCAN 2018 [38].

According to the current 11th revision of the International Classification of Diseases (ICD-11) from the World Health Organization (WHO) [37], a breast cancer (2C6) is one of approximately 100 diagnosable malignancies. The incidence and mortality trends for breast malignances are steadily growing [38]. Breast cancer is the most frequently diagnosed cancer in women, and the most common cause of death in the female population, as well as the second most common cause of death after lung cancer [39]. It accounts for around 24.2% of all cancers in the female population and about 15% of all deaths [38] (see Figure 1). Among young women (25–40 years old), the incidence and mortality rates for breast cancer are even higher and amounted to 27% and 29%, respectively. In middle-aged women (40–64 years old), these rates are 28% and 17%, and in females over 65, they are 18% and 14%, respectively [40]. In many developing countries with a high Human Development Index (HDI), such as the USA, Canada, Australia, or certain countries in the European Union [38], breast cancer is the second leading cause of death. An aging society and unhealthy lifestyles will certainly increase the number of breast cancer diagnoses in the future. For this reason, early detection, and treatment of breast cancer using modern and effective medical techniques is crucial [39].

**Figure 2:**
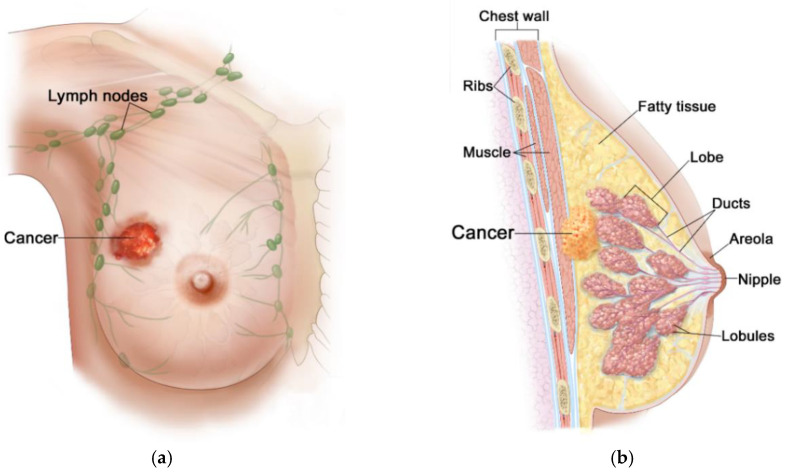
Anatomical view of female breast including tumor inside: (**a**) front view; (**b**) sectional view. Reproduced with kind permission from 2012 Terese Winslow LLC. Adapted from [44].

Generally, the female breast is made of adipose tissue, glandular tissue (including 15–20 lobules and ducts), and connective tissue (including blood, lymph, and lymph nodes), and is mounted on the pectoral muscle [41], as depicted in Figure 2. Due to rich vascularization, the female breast is an ideal environment for the development of various types of neoplastic changes. Pathological cells of the breast proliferate and abnormal mutations leading to malignancies can appear [40]. Breast carcinoma develops locally in the breast—most often in glandular tissue as flat lesions or tumor-shaped lesions. Breast tumors often have irregular shapes and may be encapsulated or not [42]. Importantly, an invasive breast cancer can spread to the lymph nodes and internal human organs, including the lungs, liver, brain, and bones. Breast density (with more glandular tissue) decreases with age. As the population ages, most of the breast glands (lobes and ducts) are replaced with fat and the breasts become less dense. Females with dense glandular tissue have a greater risk of developing breast cancer and the cancer risk increases with age (about 90% of cases are in women over 50), obesity [39], and genetic predisposition to inherited gene (BRCA1, BRCA2) mutations [43]. The most common breast cancers are pre-invasive and invasive ductal carcinoma, and lobular carcinoma, and much more rarely Paget’s cancer, sarcomas, and inflammatory breast cancer [44]. The first symptoms of malignances are changes in the structure of the breasts, thickenings, and the appearance of lumps. Breast cancer can multiply to form a hard lump or tumor, or can spread to the lymph nodes or other tissues through the circulatory or lymphatic system, forming a metastasis. What is important, during the diagnosis of breast cancer, is that noninvasive techniques for breast imaging (mammography, X-rays, CT scans, MRI scans, ultrasound exams) and invasive techniques (blood markers tests, biopsy) are commonly employed [42]. Nowadays, thanks to the widespread use of population screening tests, breast cancer is often detected in its early, asymptomatic stage, and in most cases it is completely curable [2]. The experience of many countries demonstrates that the early diagnostics and the use of a modern healing modality for breast cancer can reduce its mortality rates by up to 20–30% [40].

Knowledge of breast cancer stages is essential for planning the appropriate treatment of breast tumors [45]. Cancerous disease staging is assessed on the basis of the tumor-nodes-metastasis (TNM) classification developed by the American Joint Committee on Cancer (AJCC), which has been published continuously since 1977 [46]. On the basis of this classification, the optimal method for treating different types and locations of neoplasms causing/not causing specific metastases in nearby lymph nodes or distant organs, including the brain, lungs, liver and bones, is determined. Depending on the stage of breast carcinoma, breast tumors may be less than 2 cm in diameter (stages IA and IB), 2–5 cm (stages IIA, IIIB and IIIC), or larger than 5 cm in diameter (stages IIB, IIIA, and IV) [44]. It is worth emphasizing that the treatment of breast carcinoma depends on the stage of the cancer disease. The most common lumps develop in glandular tissue, in the upper side of the breast (35% of cases), and less often in the medial part (5%). In addition to traditional surgery, leading to tumor resection, lymph node dissection, or total breast mastectomy, the use of breast-conserving therapies, such as radiotherapy, chemotherapy, immunotherapy, targeted therapy, hormone therapy, or hyperthermia are very popular in the treatment of breast cancers [4]. In recent years, various techniques for hyperthermia treatment have been developing rapidly. Their effectiveness continues to increase, especially when combined with other anti-cancer modalities [3,16]. Combination therapies can also be utilized to induce tumor size reduction before surgery (preoperative therapies) or remove any surviving malignant cells after surgery and reduce the risk of cancer recurrence (postoperative therapies) [40].

### 2.2. Modelling

In recent years, in silico studies have become a basic tool in the analysis of various phenomena occurring in living organisms. Numerical modelling, i.e., computer-based simulation is an extremely powerful tool for solving various critical engineering and medical problems. It enables the design of new theoretical solutions without the need to conduct tests on a living organism. The basis of in silico studies for breast cancer is the utilization of adequate numerical models of the female breast in order to best reflect its anatomical structure as well as the electrical and thermal parameters of individual tissues. The first important element for accurate, realistic breast phantoms is the adoption of the appropriate geometry for the computational model. The degree of complexity of breast models available in the subject literature is very wide, ranging from basic two-dimensional (2D), homogeneous (uniform), heterogeneous (multi-tissue or multi-layered) models to very complex, anatomically correct, anthropomorphic, three-dimensional (3D) models, often based on medical imaging including CT or MRI scans. Depending on the percentage composition of the individual tissues used to build the female breast, the following models can be distinguished [6,47,48]: predominantly fatty (PF; muscle—10%, gland—20%, fat—70%), scattered fibro-glandular (SFG, muscle—20%, gland—40%, fat—40%), heterogeneously dense (HD; muscle—20%, gland—60%, fat—20%), and extremely dense (ED; muscle—20%, gland—70%, fat—10%). Such models are made up of smaller computational elements (pixels or voxels) to which various parameters of breast tissues are assigned. Bioelectromagnetic models are usually based on different numerical techniques, including the finite element method (FEM) [49], finite volume method (FVM) [50], boundary volume method (BVM) [51], finite-difference time-domain (FDTD) method [52], and others. The utilization of human anatomical models usually leads to many hours of calculations, which often disqualifies them from the optimization process, for which the calculations must be repeated many times to obtain the optimal solution. To save time and computing power, researchers often propose simplified female breast models based on the semi-ellipsoidal, semi-sphere, semi-cylindrical, or semi-anatomical objects [6,10,17,53,54]. Breast models, which are anatomically correct in terms of their external shape but without a naturalistic internal structure for breast tissues, are also common in the scientific community [55]. To validate virtual female breast models, researchers often create liquid, semi-solid, and solid physical phantoms [56,57,58,59].

**Figure 3:**
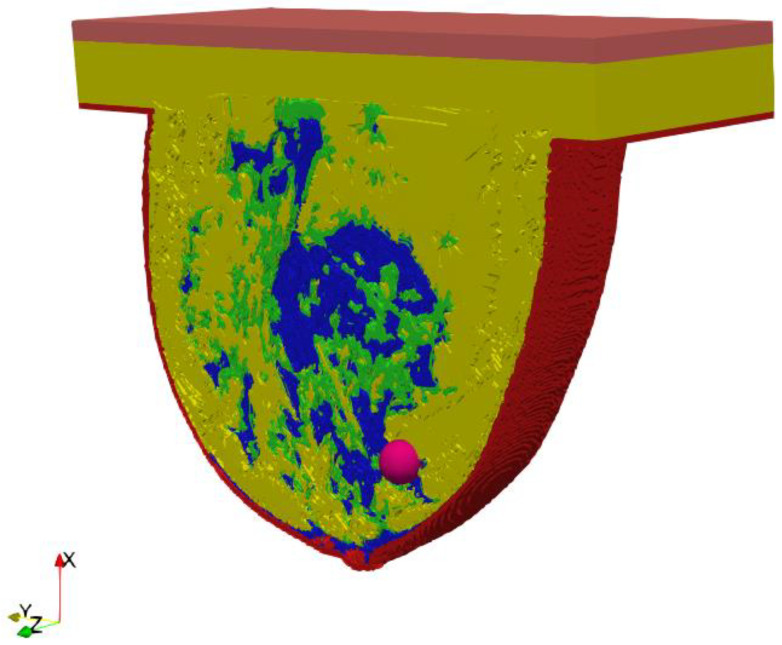
Axial cross-section (*z* = 0) through an MRI-derived breast phantom including the main breast tissues: skin (red), fat (green), breast fat (yellow), breast gland (blue), muscle (orange), tumor (pink).

A key part of this in silico study was the development of an anatomically based female breast phantom and its tissue parameters that corresponded to experimentally determined equivalents in terms of real laboratory electro-thermal measurements. The authors developed a lifelike anthropomorphic breast model based on MRI scans validated by a 3D-printed plastic breast phantom [60]. The MRI-derived breast model was characterized by a high spatial resolution of 1 mm and contained 269 × 332 × 202 cubic cells. Such a model can be characterized as a heterogeneously dense (HD) breast phantom with 51–75% glandular tissue content, which corresponds to the breast structure of a 35-year-old female patient with a tumor. In addition to breast glands (marked in blue in Figure 3) and tumor (pink), the 3D geometric female breast phantom also featured other breast tissues, such as fat (green), breast fat (yellow), skin (red), and muscle (orange). Figure 3 depicts a sectional view (*xy* slice) of the geometric model passing through the tumor (*z* = 0) seated at location (0, −12, 12) mm.

All the modelled materials are considered as homogeneous, isotropic, and lossy dielectrics. The most accurate dielectric and thermo-physical properties of female breast tissues, including the tumor tissue considered for this in silico simulation, are collected in Table 1. They were taken from the IT’IS material properties database [61] for exciting an EM frequency for the dipole antenna array equal to *f* = 1 GHz. 

It should be emphasized that the values of all key electro-thermal parameters from Table 1, discussed in Section 3, are different for various types of human tissues (e.g., blood, fat, gland, skin, muscle, tumor) and determined experimentally in [62,63]. It was assumed that the breast tumor had breast muscle tissue properties with different heat transfer rates (HTRs). The temperature-dependent HTR parameter in a tumor is due to the specific dense vascularity and blood perfusion of breast carcinoma [52,64]. It is worth adding that the electrical parameters such as relative permittivity (*ε*_r_) and electrical conductivity (σ) strongly depend on the frequency of the external EM field produced by the matrix of the dipole antennas. This is due to the dispersion properties of human tissues and tissue relaxation processes occurring under the influence of an alternating EM field [62]. In our case, the temperature-dependence of breast parameters was omitted excluding the tumor blood perfusion defined in Equation (3) as a non-linear quantity.

**Figure 4:**
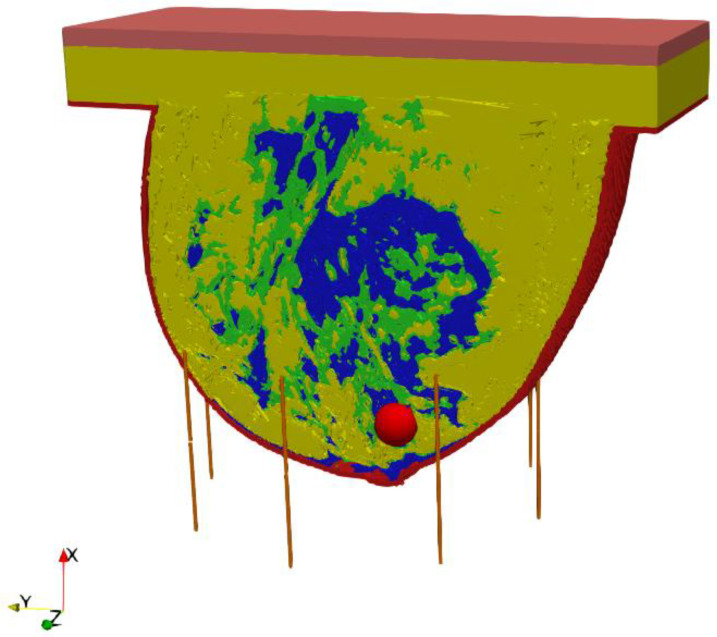
The model of eight element dipole antenna array surrounding the female breast phantom.

The entire model, including the female breast tissues with embedded tumor and dipole antenna array, consisted of 265 × 379 × 259 voxels of a volume 1 mm^3^ each. The considered antennas matrix included eight dipole antennas forming an array with diameter of 10 cm surrounding the female breast model, as presented in Figure 4. All the dipole applicators placed around the breast were powered at the same frequency of 1 GHz, but each dipole could be individually tuned for amplitude and phase so that the appropriate power deposition could be focused on the treated tumor region or ROI, avoiding healthy tissues. The problem was solved using the Cartesian coordinate system (*x*, *y*, *z*), and the center of the dipole array was taken as a reference point (0, 0, 0).

The Maxwell’s equations [17] together with the modified Pennes’ bioheat model (Equation (1)), described in detail in Section 3 which follows, are supplemented by suitable boundary and initial conditions. The dipole antennas were considered to be perfect electric conductors (PECs). The computational domain was bounded by six perfectly matched layers (PMLs)—two in the direction of each axis, which leads to undistorted penetration of the EM field beyond the computing area. In order to solve the electro-thermal formulations, we applied the FDTD method using the commercially available Sim4Life software [36]. The dipole antennas’ exposure time (at the time of tumor treatment), appearing in the modified Pennes’ bioheat model (Equation (1)), was assumed to be 30 min.

**Figure 5:**
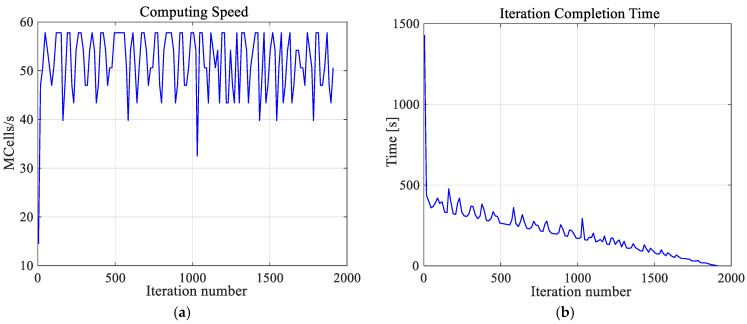
The computing speed (**a**) and iteration completion time (**b**) as function of iterations number for the thermal solver. The convergence was obtained after 1911 iterations.

It is worth emphasizing that the high-resolution numerical model of a female breast, created by us, was achieved at great computational cost. The calculations were undertaken using a dual processor workstation Intel(R) Xenon(R) CPU E5-26090 2.4 GHz, 2.4 GHz with 64 GB RAM. The hardware was additionally accelerated using the latest high-performance computing technology, which is dedicated and optimized for FDTD solver, i.e., CUDA with graphics card NVIDIA Tesla C2075. Using this approach, the simulation of one case (optimization for one exemplary tumor size) took approximately 2 h and 40 min and was about three times than it would have taken to solve the model without acceleration. Additionally, Figure 5 depicts the thermal solver computing speed and the number of iterations of the thermal solver. The temperature in the analyzed model was determined after 23 min and 50 s with the speed of 51.4 × 10^6^ cubic cells per second in one iteration. The convergence after 1911 iteration was obtained.

## 3. Results and Discussion

Using the described optimization procedure, the input power levels (*P_i_*) together with the phases of the dipole antennas (*ϕ_i_*) for individual applicators were successfully determined. It should be emphasized that the values for optimal *P_i_* and *ϕ_i_* parameters are summarized in Table 2 for different tumor radii *r* growing incrementally from 2 mm to 12 mm, in steps of 2 mm. Moreover, the total MW power of all the antennas was found to be 8 W, which directly translates into the expected breast cancer temperature of about 42 °C.

**Figure 6:**
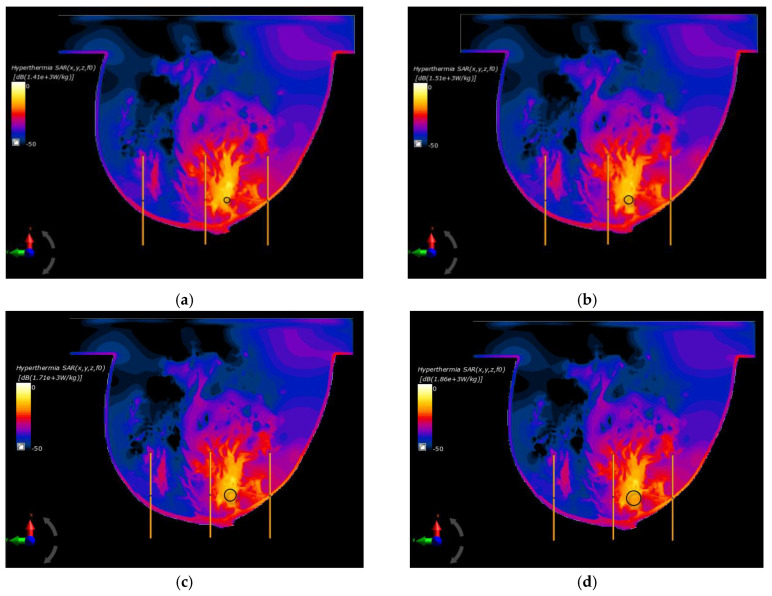

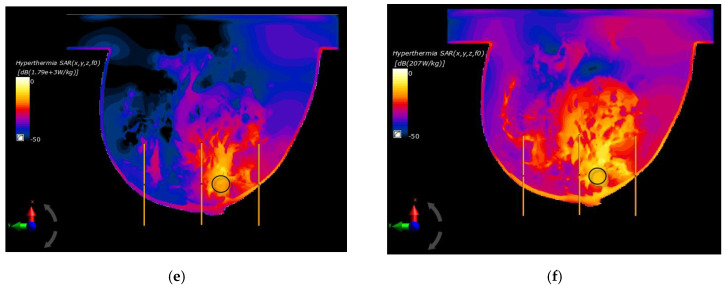
Normalized specific absorption rate (SAR) distributions in the *xy* plane from an eight-dipole antenna array after the optimization procedure (sum *P_i_* = 8W) for various tumor radii: (**a**) *r* = 4 mm; (**b**) *r* = 6 mm; (**c**) *r* = 8 mm; (**d**) *r* = 10 mm; (**e**) *r* = 12 mm; (**f**) *r* = 12 mm—before the optimization procedure (*ϕ_i_* = 0 and *P_i_* = 1 W).

In the last part of this paper, the optimal parameters, gathered in Table 2, were used to specify the SAR distributions as well as the radius-dependent thermal profiles of spherical breast cancers. In this case, Figure 6 shows the normalized SAR distributions in the *xy* plane for steady state inside the breast models with various tumor diameters surrounded by the matrix of dipole antennas working at optimal *P_i_* and *ϕ_i_* parameters (sum *P_i_* = 8 W). All the distributions were normalized to the maximum SAR value and were expressed on a decibel scale. As can be seen, the maximum SAR value in the tumor increased as its size increased, reaching values of SAR_max_ = 1.41, 1.51, 1.71, 1.79, and 1.86 kW/kg for tumor radii *r* = 4, 6, 8, 10, and 12 mm, respectively.

Figure 6f shows an example of SAR distribution for a tumor radius *r* = 12 mm; however, this was before applying the optimization algorithm (all antennas operate with the same *P_i_* and *ϕ_i_* values). In this case, the SAR values within the breast tissues around the applicators were similar and had values below 207 W/kg, which illustrates the advantage of warm colors. On the other hand, with optimized SAR distributions, warm colors were concentrated in the immediate vicinity of the breast cancer, which corresponds to an increased power dissipation and heat accumulation in this area.

**Figure 7:**
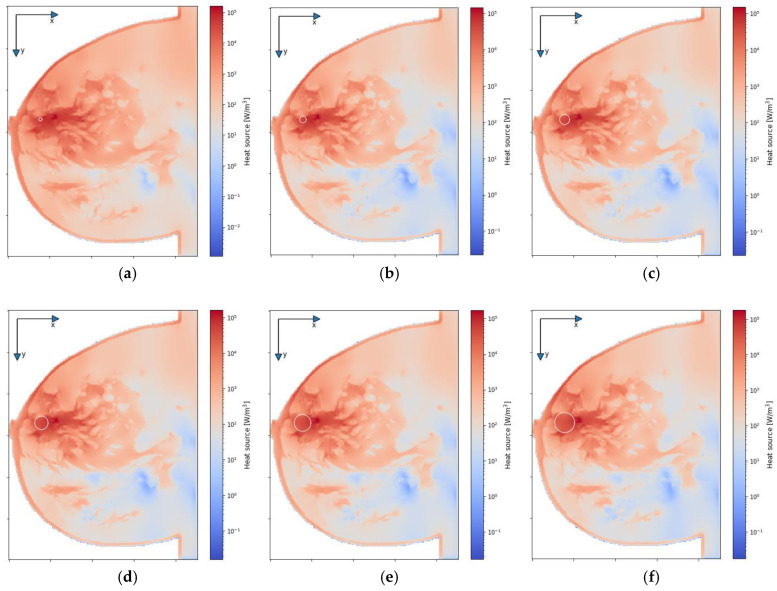
The distributions of power dissipation (*Q* = *ρ*SAR) in the *xy* plane for various tumor radii: (**a**) *r* = 2 mm; (**b**) *r* = 4 mm; (**c**) *r* = 6 mm; (**d**) *r* = 8 mm; (**e**) *r* = 10 mm; (**f**) *r* = 12 mm—derived from the eight-dipole antenna array after the optimization procedure (sum *P_i_* = 8 W).

**Figure 8:**
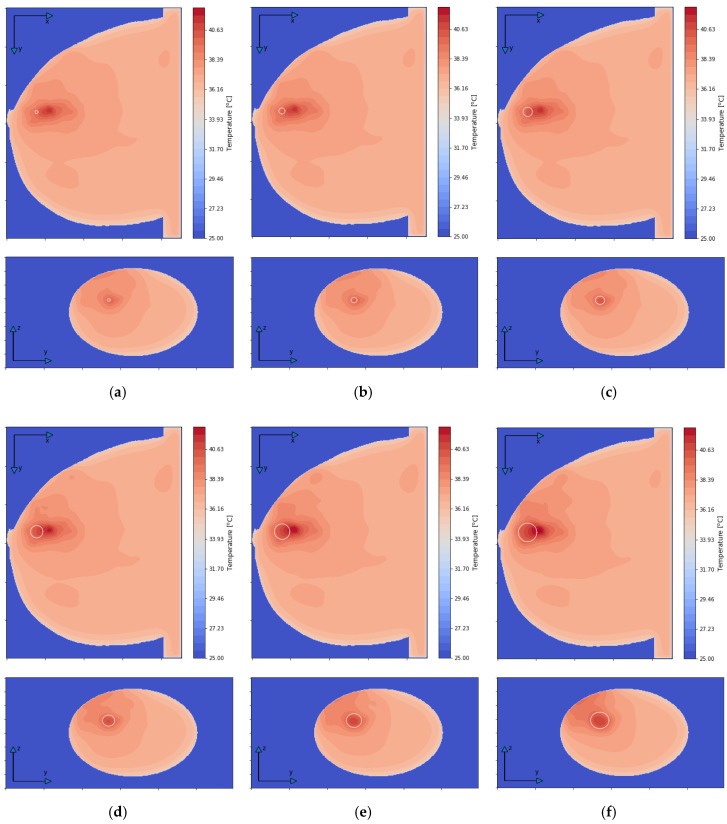
The temperature distributions in the *xy* and *zy* planes for various tumor radii: (**a**) *r* = 2 mm; (**b**) *r* = 4 mm; (**c**) *r* = 6 mm; (**d**) *r* = 8 mm; (**e**) *r* = 10 mm; (**f**) *r* = 12 mm—derived from the eight-dipole antenna array after the optimization procedure (sum *P_i_* = 8 W).

Figure 7 depicts the distributions for the heat source (*Q* = *ρ*SAR) in the *xy* plane of the breast model passing through the tumor center (*z* = 0) after the optimization procedure. Similar distributions for temperature are presented in Figure 8. Additionally, the cross sections in the *zy* plane for the female breast phantom passing through the tumor center (*x* = 0) are included in Figure 8. It should be noted from the given drawings that the optimization algorithm used is good at dealing with tumors of various sizes. However, for larger tumors with radii greater than *r* = 6 mm, its reliability is much better. 

**Figure 9:**
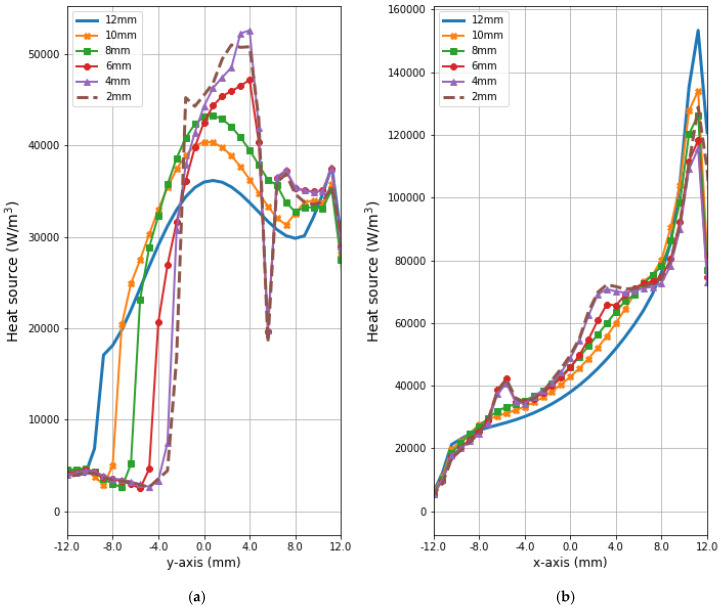
The distributions of power dissipation (*Q* = *ρ*SAR) running through the center of tumors with different sizes along (**a**) the *y*-axis perpendicular to the antenna matrix; (**b**) the *x*-axis in the direction of the eight-dipole antenna array after the optimization procedure.

**Figure 10:**
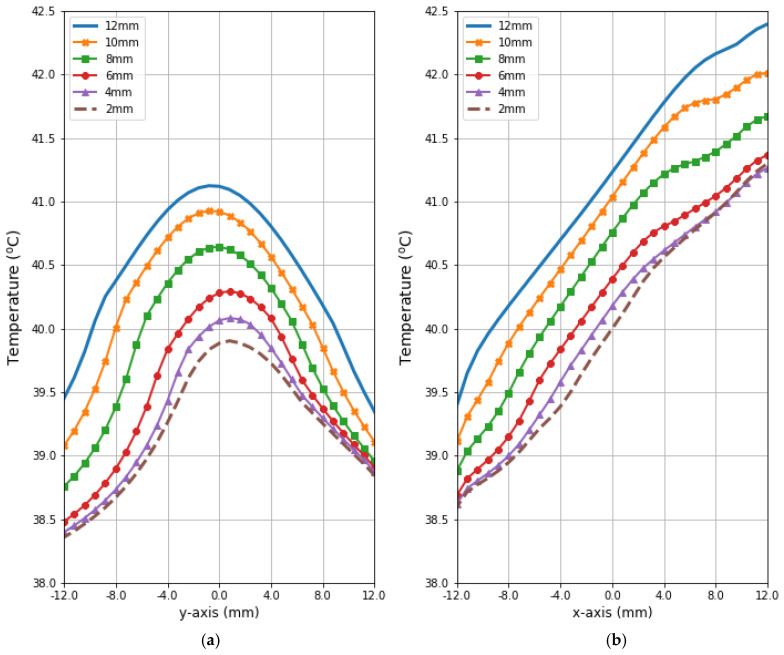
The heating curves running through the center of tumor of different sizes along (**a**) the *y*-axis perpendicular to the antenna matrix; (**b**) the *x*-axis in the direction of the eight-dipole antenna array after the optimization procedure.

Figure 9 and Figure 10 present the heat source (*Q* = *ρ*SAR) and temperature dependencies through the tumor along the *y*- and *x*-axes, respectively. All curves are referred to the tumor center and compare the distributions for tumors of different sizes. Larger changes in the analyzed parameters are observed along the *x*-axis than the *y*-axis of the tumor, which results from the arrangement of various tissues around breast cancer and different perfusion rates characterizing breast tissues. Additionally, the character of *Q*-profiles strongly depends on the density of the tumor and neighboring tissues. This implies that homogeneous temperature distribution inside the whole tumor volume cannot be achieved in practice by using the described optimization procedure. However, a specific carcinoma’s behavior can be seen in the performed in silico simulation, namely, for tumors of larger size, higher temperature levels are induced in the tumor.

**Figure 11:**
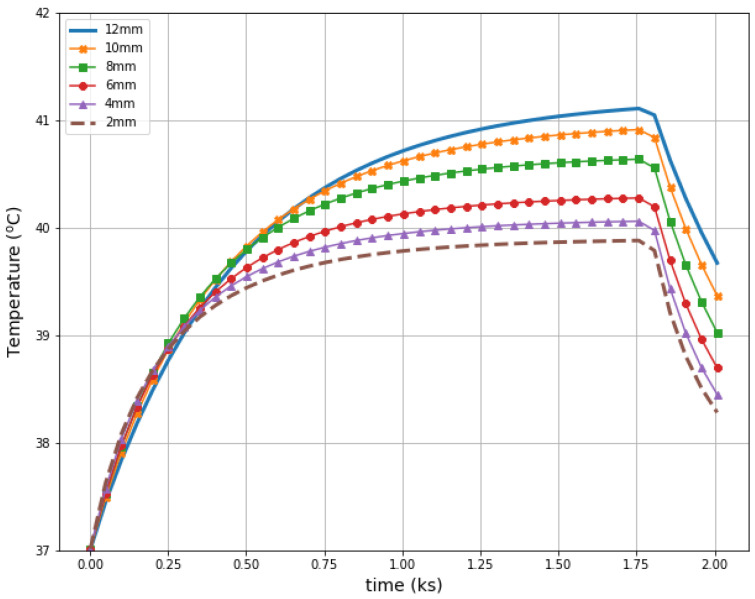
The transient temperature distributions inside the tumors subjected to the eight-dipole antenna array after the optimization procedure.

Figure 11 illustrates radius-dependent heat accumulation inside the analyzed breast tumors, according to the modified Pennes’ bioheat equation (Equation (1)). The obtained temperatures in the tumor centers, in the transient state, varied from 39.9 to 41.1 °C, and thus they did not exceed therapeutic levels for hyperthermia treatment. After a 30-min period, the dipoles were turned off, which was illustrated by the collapse of the trend to increase in the breast tumor heating curves.

## 4. Materials and Methods

Mathematical modeling of hyperthermic heating of pathological breast tissues was based on a numerical solution of an electro-thermal-coupled problem, as presented in the following subsections.

### 4.1. The Electric Field from the Dipole Antenna

The electro-thermal interaction of female breast tissues affected by the tumor with microwaves produced by a circular array of dipole antennas was solved using the finite-difference time-domain (FDTD) engine relying on the Maxwell’s equations in a time domain, as described in [17]. Details of the FDTD paradigm based on the cubic cells can be found in several critical studies [20,25,65].

**Figure 12:**
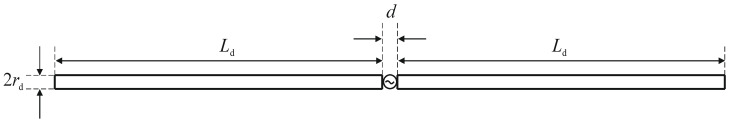
View of the dipole antenna including dimensions (*d* = 1 mm, *L*_d_ = 50 mm, *r*_d_ = 1 mm) and the sinusoidal voltage dipole source.

**Figure 13:**
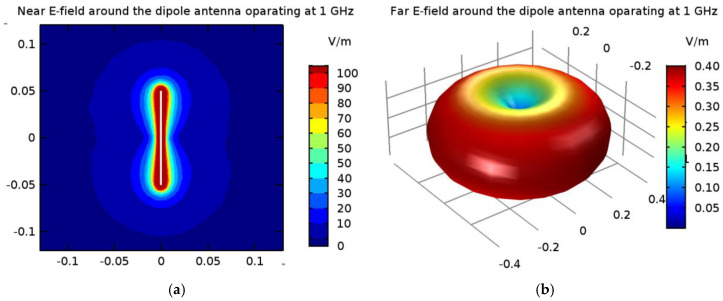
The electric field derived from the 1-GHz half-wave dipole antenna with a shortening ratio set at *k*_s_ = 0.67: (**a**) near field distribution; (**b**) far field radiation.

A matrix of 8 dipole antennas arranged symmetrically around the female breast phantom was used as the source of EM excitation (see Figure 4). Due to limited space around the analyzed breast tissue, the shortened-length, half-wave dipole antennas with a shortening factor established at value *k*_s_ = 0.67 were employed in the model. Therefore, each antenna of the array operated at a working frequency of 1 GHz and consisted of two symmetrical dipole arms of length *L*_d_ = 50 mm and radius *r*_d_ = 1 mm, as well as an AC voltage source *e_i_*(*t*) = *A*_m_cos(2π*ft* + *ϕ*_i_) supplying the dipole, as shown in Figure 12. Importantly, a dipole source induces currents flowing in opposite directions on the surface of the metal dipole arms. In turn, these currents cause the antenna radiation and the *E*-field distribution around the dipole (see Figure 13).

In the popular FEM- and FDTD-based engines, the dipole antenna is modelled as a two-armed metal antenna with a perfect electric conductor (PEC). Thus, the zero value of the tangent component of the electric field strength (*E_x_* = 0) is assumed on the surface of each dipole arm. Generally, a symmetrical transmission line with characteristic impedance of 50 Ω supplies a dipole antenna. For numerical calculation purposes, modeling of the feeding coaxial cable is omitted. It is sufficient that each dipole is fed by a point-dipole sinusoidal voltage source with an internal load of 50 Ω, located between the dipole arms. In the FDTD-based software Sim4Life [36], this was modelled using the edge port of length *d* = 1 mm (*d* << *λ*) with a time-harmonic excitation signal defined by the amplitude *A*_m_ and phase *ϕ_i_* of the dipole source. It was assumed that the direction of this source was in opposition (i.e., in the *x*-direction) to the resulting vector of electric field strength **E** = *E_x_***e*_x_*** between the antenna arms, and that its amplitude was equal to *A*_m_ = 1 V. Moreover, the initial input power values for the dipole sources were set at *P_i_* = 1 W, and thus we were freely able to set the crucial parameters of the dipole antennas *P_i_* and *ϕ_i_*, while the amplitude *A*_m_ and the frequency *f* of dipole sources did not change. By changing of the antenna frequency *f*, and thus the wavelength *λ* = *c*/*f*, where *c* = 3∙10^8^ m/s is the speed of light, we were able to influence the shape of the dipole antennas’ radiation characteristics. Obviously, a higher frequency is related to a shorter wavelength and thus a smaller size of dipole antenna. In the case of the analyzed shortened 1-GHz-half-wave applicator, the *E*-field characteristics around a single dipole are depicted in Figure 13.

**Figure 14:**
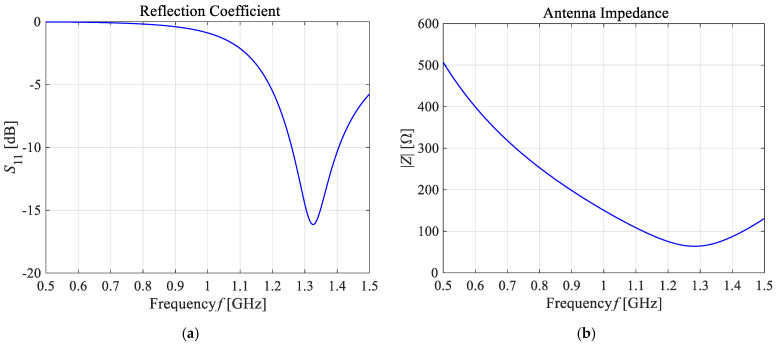
The main frequency-dependent characteristics of the shortened 1-GHz half-wave dipole antenna: (**a**) the reflection coefficient (*S*_11_ = −16.11 dB); (**b**) the input impedance (*Z*_in_ = 63.8 Ω).

As presented in Figure 13, the greatest values for the *E*-field appear around the excitation source and the ends of the dipole antenna arms. For better visibility, the near field dipole distribution scale has been limited to the value of *E* = 100 V/m. Importantly, the *E*-maxima observed near the antenna have no effect on the field distribution at a large distance from the antenna where the field is quasi-homogeneous and the radiation pattern is isotropic. In the far-field (known as the antenna radiation zone), the electric field from a single dipole antenna is a plane wave. The modeled shortened 1-GHz half-wave dipole antenna is an the omnidirectional antenna, i.e., the waves emitted by the antenna propagate with the same intensity in each direction, the radiation pattern is torus-shaped, and the *E*-field disappears along the *x*-axis of the dipole antenna. Two of the most important characteristics of the used applicator are shown in Figure 14, i.e., the reflection coefficient and the input impedance of the dipole antenna. The definitions of these quantities are detailed described in [7]. Moreover, the characteristic extremes are visible for the resonant frequency of about 1.3 GHz (*S*_11_ = −16.11 dB and *Z*_in_ = 63.8 Ω), as seen in the curves in Figure 14. It is worth emphasizing that a resonant frequency of the single dipole antenna differs from the 1-GHz antenna operating frequency, which is an inevitable consequence of shortening the dipole antenna size.

From the viewpoint of the discussed case of hyperthermia for female breast tissues affected by a tumor and surrounded by a matrix of 8 dipole antennas, we were particularly interested in the near field to determine the specific absorption rate (SAR) and, in effect, the breast tumor temperature.

### 4.2. The Modified Pennes Bioheat Transfer Model

The most common model describing the heat exchange inside biological objects in macroscopic terms is the Pennes’ heat transfer model derived from Fourier’s heat conducting equation in 1948 [66]. The classic Pennes’ equation took into account two biologically relevant heat sources reflecting the cooling effect of blood flowing in the arterial and venous blood vessels, as well as tissue metabolism. To estimate the thermal characteristics of female breast tissues in building the analyzed phantom, researchers successfully employed the modified Pennes’ bioheat equation in the following form [63,67]:

(1) $$\rho C\frac{\partial T}{\partial t}+\nabla \cdot \left(-k\nabla T\right)=\rho_{b}C_{b}\rho \mathrm{HTR}\left(T_{b}-T\right)+\rho \mathrm{HGR}+\rho \mathrm{SAR}$$

The first term of the above equation describes the phenomenon of heat accumulation in breast tissues during treatment time *t* [s], which is specified by such tissue parameters as the specific heat *C* (J/kg/K) and mass density *ρ* (kg/m^3^). The second element relates to the heat conduction phenomenon inside each tissue, which is significantly influenced by the tissue’s thermal conductivity *k* (W/m/K). The third element describes the heat exchange between the individual tissues and arterial blood and determines the heat loss caused due to the cooling effect of blood flow through the tissue (commonly known as perfusion). These losses are proportional to the heat transfer rate HTR (mL/min/kg) and the temperature difference between arterial blood (*T*_b_) and tissue (*T*) [66]. The last two elements of the modified Pennes’ equation apply to heat generation due to the metabolic activity of tissue dependent on the heat generation rate (HGR; W/kg) as well as due to external EM sources (in our case, multi-element dipole antenna array) based on specific absorption rate (SAR; W/kg) defined in Equation (2). The last element *Q* = *ρ*SAR (W/m^3^), commonly called external heat generation, relates to the coupling to the EM field, and from a theoretical point of view, it is a volumetric power density. Notably, the effective SAR parameter as a measure of the EM energy deposited by the unit mass of the biological objects a under unit period of time is written as follows [17]:

(2) $$\mathrm{SAR}=\frac{d}{dt}\left(\frac{dW}{dm}\right)=\frac{d}{dt}\left(\frac{dW}{\rho dV}\right)=\frac{\sigma }{2\rho }{\left|E\right|}^{2}=\frac{\sigma }{2\rho }E\cdot E^{*}\sim \frac{dT}{dt}$$

where *W* (J) refers to the energy absorbed by the breast tissue phantom with a specified volume *V* (m^3^), mass m (kg), and mass density *ρ* (kg/m^3^). Moreover, |**E**| (V/m) corresponds to the magnitude of the total electric field produced by the antenna array as the superposition of each dipole, *σ* (S/m) stands for electrical conductivity of the body, and *t* (s) is the time of EMF exposure. Importantly, the SAR value is reflected in the increase in temperature distribution for the breast tissue *T* (K), as shown in the modified Pennes equation (Equation (1)). Therefore, the measurement and monitoring of this parameter may play an important role in terms of establishing EM hazards and human tissue safety [25,68,69,70].

The blood perfusion rate inside the tumor was assumed to be highly non-linear in terms of temperature dependence to reflect both the dense and dysfunctional vascularization of cancerous tissue as well as the complex bio-regulatory processes in the breast carcinoma [64], namely,

(3) $$\omega_{\mathrm{tumor}}\left(T\right)=\rho_{b}\rho \mathrm{HTR}=0.4+0.4\exp\left(-\frac{{\left(T-37\right)}^{4}}{880}\right)\left[\frac{\mathrm{kg}}{m^{3}s}\right]$$

The heat transfer rate (HTR) was assumed to be constant within the remaining female breast tissues, as indicated in Table 1. As demonstrated in our previous study [64], the value of the ω parameter at a hyperthermic therapeutic temperature of 42 °C fully corresponds to the linear and constant blood flow models inside the tumor.

The modified Pennes’ bioheat model (Equation (1)) should also be supplemented with adequate initial and boundary conditions (see Equation (4)). The Robin-type boundary condition was employed on the skin surface of the modelled breast phantom in order to simulate natural convection from the human body to the external environment, according to the formula [52]:

(4) $$n\cdot \left(k_{\mathrm{skin}}\nabla T\right)=h\left(T-T_{ext}\right)$$

where *h* (W/m^2^/K) denotes the effective heat transfer coefficient specific for convection, radiation, and evaporation heat losses; *k*_skin_ (W/m/K) refers to the thermal conductivity of the breast skin layer; *T*_ext_ = 25 °C is both the ambient room temperature and temperature of the breast model exterior; and **n** is the unit vector normal to the surface of the breast skin. Additionally, on the external boundaries of the computational domain, a thermal insulation is assumed. The arterial blood temperature was set at the level *T*_b_ = 37 °C, which is equivalent to a baseline physiological body core temperature for the human body. The initial breast temperature was considered to be *T*_0_ = *T*_b_ = 37 °C.

### 4.3. Field from Annular Dipole Antenna Array and Optimization Procedure

**Figure 15:**
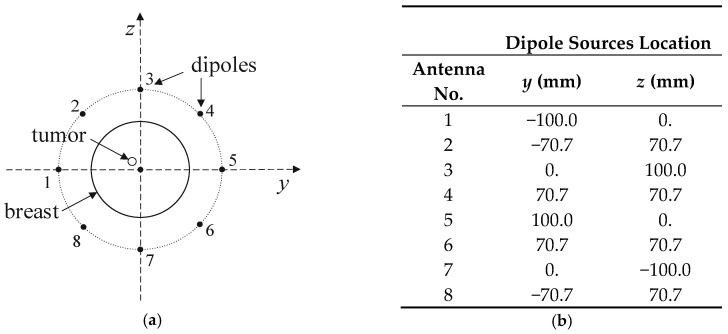
Arrangement of dipole antennas around the breast with a tumor of radius *r* = 2 mm centered at (0, −12,12) mm: (**a**): schematic sectional view; (**b**): dipole sources positions at *x* = 0 plane (antenna array radius *R* = 100 mm).

The considered hyperthermia system is formed using a circular array of 8 dipole antennas, each with a radius of *R* = 100 mm surrounding an anatomically correct female breast phantom as schematically viewed in Figure 15. The authors chose the antenna array radius arbitrarily to accommodate female breasts of different sizes inside the applicator. The dipoles were evenly distributed around the breast model on the *yz* plane and their sources were localized in the *x* = 0 plane and numbered. The antenna array position was chosen with regard to the antennas feeding points and the tumor center in order to achieve symmetrical tumor exposition. Before the optimization procedure, all dipole sources were running at the same frequency of *f* = 1 GHz, unit amplitudes of *A*_m_ = 1 V, and zero phases *ϕ_i_*. Moreover, initially all applicators worked with the same input power level, set at *P_i_* = 1 W, and the whole eight-element dipole array operated with a correspondingly higher MW power. The limitation of the total input power of the antenna array to the sum *P_i_* = 8 W was derived from our earlier studies [17] as the best option to obtain healing temperature levels for hyperthermia, i.e., 42 °C.

**Figure 16:**
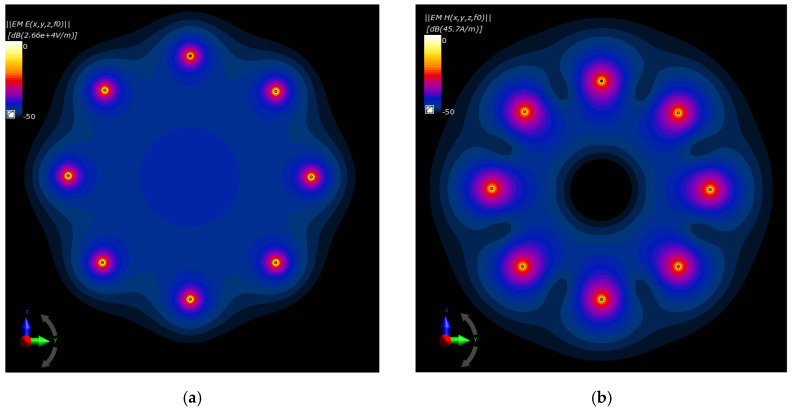
Normalized electric and magnetic field distributions from the antenna array when each dipole is working at *f* = 1 GHz and *P_i_* = 1 W before the optimization procedure: (**a**) *E*-field; (**b**) *H*-field.

The drawings compared in Figure 16a,b depict, respectively, the electric and magnetic field distributions generated by the analyzed antenna matrix before optimization. To better illustrate the EM field in the ROI, we normalized the *E*- and *H*-field distributions to the maximum value and were given on a decibel scale. As expected, the same initial set points for the dipole sources resulted in symmetrical field distribution around the dipoles and homogeneous field distribution inside the antenna array. Only proper parameter optimization of the dipole antenna array will allow for EM energy deposition in the target tissue and thereby heat the breast cancer to healing temperatures.

The EM energy deposition in the analyzed breast tissues with an immersed tumor is determined numerically for each dipole antenna applicator. The electric field **E** (V/m) generated by a single dipole antenna supplied by voltage with a unit amplitude and no phase shift, at a specific location in the space given by a distance vector **r** = *x***e***_x_* + *y***e***_y_* + *z***e***_z_*, is expressed by the following formula [19]:

(5) $$E_{n}(r)=\sum_{i}^{x,y,z}E_{n,i}(r)\exp\left[j\Phi_{n,i}(r)\right]e_{i}$$

where *E_n,I_* (V/m) and Φ*_n,I_* (rad) are, respectively, the amplitude and the phase shift of the resulting electric field strength of the *n*-th applicator for each *i*-th orthogonal component of the Cartesian coordinate system (*x*,*y*,*z*) and given location. Moreover, **e***_i_* represents the unit vectors toward the individual axis of the rectangular coordinate system and j = √(−1) is the imaginary unit.

In a general case where the amplitude and the phase of the *n*-th dipole antenna are *A_n_* and *ϕ_n_*, respectively, then the effective *E*-field at the current point **r** (see Equation (5)) affected by the element matrix of *N* = 8 dipole antennas is established by

(6) $$E\;(A_{1},\;\;\ldots \;\;,A_{N},\;\phi_{1},\;\;\ldots \;\;,\phi_{N},r)=\sum_{n\;=\;\;1}^{N}A_{n}\exp(j\phi_{n})\;\;\cdot \sum_{i}^{x,y,z}E_{n,i}(r)\exp\left[j\Phi_{n,i}(r)\right]\;e_{i}$$

In the following optimization procedure, it is necessary to determine the specific absorption rate SAR (W/kg) used in the modified Pennes’ bioheat transfer model (Equation (1)). By combining Equations (2) and (6), one can obtain the resultant value for the SAR parameter at the given location derived from the *N*-element dipole antenna array, i.e.,

(7) $$\mathrm{SAR}\;(A_{1},\;\;\ldots \;\;,A_{N},\;\phi_{1},\;\;\ldots \;\;,\phi_{N},r)=\sum_{n\;=\;\;1}^{N}\sum_{m\;=\;\;1}^{N}A_{m}A_{n}\frac{\sigma }{2\rho }\sum_{i}^{x,y,z}E_{m,i}(r)E_{n,i}(r)\cos\left[\Phi_{n,i}(r)-\Phi_{m,i}(r)+\phi_{n}-\phi_{m}\right]\;$$

Therefore, the SAR coefficient obtained from the *N* = 8 dipole antenna array is the sum of *N*^2^ = 64 terms for each of the spatial components of the **E**-field vector (*E_x_*, *Ey*, *Ez*). Then, using the following substitution,

(8) $$\sum_{i}\alpha_{i}\cos\left(\omega_{i}+\phi \right)=\beta \cos\left(\Omega +\phi \right)$$

where

(9) $$\beta =\sqrt{{\left(\sum_{i}\alpha_{i}\cos\left(\omega_{i}\right)\right)}^{2}+{\left(\sum_{i}\alpha_{i}\sin\left(\omega_{i}\right)\right)}^{2}}$$

and

(10) $$\Omega =\arctan\left[\frac{\sum_{i}\alpha_{i}\sin\left(\omega_{i}\right)}{\sum_{i}\alpha_{i}\cos\left(\omega_{i}\right)}\right]$$

yields the SAR definition that depends from two spatial-dependent parameters but which at the same time is independent of the dipole antennas’ amplitudes and phases, namely,

(11) $$\mathrm{SAR}\;(A_{1},\;\;\ldots \;\;,A_{N},\;\phi_{1},\;\;\ldots \;\;,\phi_{N},r)=\sum_{m\;=\;\;1}^{N}\sum_{n\;=\;\;1}^{N}A_{m}A_{n\;}\beta_{mn}(r)\cos\left[\Omega_{mn}(r)+\phi_{n}-\phi_{m}\right]\;$$

with

(12) $$\beta_{mn}(r)=\frac{\sigma }{2\rho }\sqrt{{\left(\sum_{i}^{x,y,z}E_{m,i}(r)E_{n,i}(r)\cos\left[\Phi_{n,i}(r)-\Phi_{m,i}(r)\right]\right)}^{2}+{\left(\sum_{i}^{x,y,z}E_{m,i}(r)E_{n,i}(r)\sin\left[\Phi_{n,i}(r)-\Phi_{m,i}(r)\right]\right)}^{2}}$$

and

(13) $$\Omega_{mn}(r)=\arctan\left[\frac{\sum_{i}^{x,y,z}E_{m,i}(r)E_{n,i}(r)\cos\left[\Phi_{n,i}(r)-\Phi_{m,i}(r)\right]}{\sum_{i}^{x,y,z}E_{m,i}(r)E_{n,i}(r)\sin\left[\Phi_{n,i}(r)-\Phi_{m,i}(r)\right]}\right]$$

In the optimization procedure for amplitude- and phase-dependent power deposition inside the volume of interest, it is necessary to establish the average SAR value for such volume, defined as

(14) $${\mathrm{SAR}}_{\mathrm{vol}}=\frac{1}{V}\int_{V}\mathrm{SAR}\;dV$$

which after discretization and once Equation (11) is taken into account gives the following relation:

(15) $${\mathrm{SAR}}_{\mathrm{vol}}\;(A_{1},\;\;\ldots \;\;,A_{N},\;\phi_{1},\;\;\ldots \;\;,\phi_{N})=\frac{1}{M}\sum_{m=\;\;1}^{N}\sum_{n\;=\;\;1}^{N}\sum_{l\;=\;\;1}^{M}A_{m}A_{n\;}\beta_{mn}(r_{\;l})\cos\left[\Omega_{mn}(r_{\;l})+\phi_{n}-\phi_{m}\right]\;$$

where *M* is the number of volumetric cells into which the selected volume *V* was discretized. *M* can vary from several to several thousand cells, depending on the volume of *V*. The equivalent equation with parameters independent of the coordinates take the following form:

(16) $${\mathrm{SAR}}_{\mathrm{vol}}\;(A_{1},\;\;\ldots \;\;,A_{N},\;\phi_{1},\;\;\ldots \;\;,\phi_{N})=\frac{1}{M}\sum_{m=\;\;1}^{N}\sum_{n\;=\;\;1}^{N}A_{m}A_{n\;}{\beta^{\prime }}_{mn}\cos\left[{\Omega^{\prime }}_{mn}+\phi_{n}-\phi_{m}\right]\;$$

where

(17) $${\beta^{\prime }}_{mn}=\sqrt{{\left(\frac{1}{M}\sum_{l\;=\;\;1}^{M}\beta_{mn}(r_{\;l})\cos\left[{\Omega^{\prime }}_{mn}(r_{\;l})\right]\right)}^{2}+{\left(\frac{1}{M}\sum_{l\;=\;\;1}^{M}\beta_{mn}(r_{\;l})\sin\left[{\Omega^{\prime }}_{mn}(r_{\;l})\right]\right)}^{2}}$$

and

(18) $${\Omega^{\prime }}_{mn}=\arctan\left[\frac{\sum_{l\;=\;\;1}^{M}\beta_{mn}(r_{\;l})\sin\left[{\Omega^{\prime }}_{mn}(r_{\;l})\right]}{\sum_{l\;=\;\;1}^{M}\beta_{mn}(r_{\;l})\cos\left[{\Omega^{\prime }}_{mn}(r_{\;l})\right]}\right]$$

It is necessary to emphasize that Equation (16) does not depend on the location (spatial-dependent parameters) or the amplitude and the phase of individual antennas in the matrix, but only on the specific excitation parameters of the dipole antennas, namely, *β’_mn_* and Ω*’_mn_*. Moreover, this reduces the necessary computing power and shortens the calculation time.

The presented approach (see Equations (5–18)) allows for a multi-objective optimization problem for the SAR ratio of two selected volumes of interest and optimal heating of only one of them. In the analyzed case of the breast model affected by cancer, the purpose was to heat the tumor region most effectively, while limiting the heating of other female breast tissues. This goal can be achieved by fulfillment of the following objective function [17,35]:

(19) $$\max\;{\mathrm{SAR}}_{\mathrm{ratio}}\;=\max\;\frac{{\mathrm{SAR}}_{\mathrm{vol tumor}}}{{\mathrm{SAR}}_{\mathrm{vol breast}}}=\max\;\frac{\int_{\mathrm{tumor}}w\left(r\right)\mathrm{SAR}\left(r\right)dV}{\int_{\mathrm{breast}}w\left(r\right)\mathrm{SAR}\left(r\right)dV}$$

where *w* denotes the relevant weighting factors. In our optimization procedure, it was assumed that all *w* parameters have unit initial values.

In the next step, the optimal input powers and phase values for individual dipoles that meet the Equation (19) were specified. The Sim4Life in-built optimization procedure assumes that all dipole antennas in the array are supplied independently, and that there is no coupling between their feeding cables, and thus it is not possible to determine their mutual transmission coefficients *S_ij_* (*i* ≠ *j*), as defined in [7]. Additionally, the given optimization procedure assumes that the phases of the dipole sources *ϕ_i_* range from −180° to 180° and the total MW power of the entire dipole antenna array is equal to the sum *P_i_* = 8 W, with the initial values for all the applicators as follows: *ϕ*_0_ = 0 and *P*_0_ = 1 W. All the amplitudes for dipole sources have unit values of *A_i_* = 1 V. A similar procedure for a two-spherical object has been briefly described in [17]. The inverse form of such an objective function (19) based on a desired frequency bandwidth has been shown in [71]. It is important to note that recently, some novel energy/SAR-focusing optimization techniques have been investigated in the field of hyperthermia treatment, including *E*-field focusing via constrained power optimization (FOCO) [34], the *E*-field focusing [21], the time reversal focusing method [23], and others [19]. As demonstrated in paper [34], the FOCO approach gives slightly better results compared to the clinical benchmark using particle swarm-based optimization (PSO), especially for larger volumes of treated regions. A similar study [18] proposes a multi-frequency-based phased array with 8 monopole antennas for targeted hyperthermia treatment of deep-seated and multi-locus carcinomas in the head and neck. The conclusion was that multi-frequency excitation improves SAR accumulation.

## 5. Conclusions

Numerical modeling of localized heating of a tumor embedded within an anatomically accurate female breast model using an eight-dipole antennas array, with the given optimization procedure, offered satisfactory results, regardless of the tumor size. Due to the dimension limitations around a female breast, we selected the 1-GHz shortened-length dipole antennas for the EM excitations, whose phases and powers were changed while maintaining an unchanged power of 8 W for the entire dipole matrix. The FDTD-based in silico simulation provides a reliable numerical solution of described electro-thermally problem and produces satisfactory thermal tumor profiles at the level of 40–41 °C for analyzed tumors of different sizes. Importantly, the considered model includes the non-linear temperature dependence of the tumor perfusion, which allows for a more realistic estimation of tumor temperature. Increasing the tumor temperature above the physiological temperature of 37 °C was found to significantly reduce perfusion in the tumor, which, in turn, caused even greater heat accumulation in the tumor when conducting cancer-localized MW hyperthermia. This investigation demonstrates that optimal thermal tumor profiles depend on the radius of the modelled tumor. The numerical calculation findings indicate that the increased tumor diameters caused higher temperature increments within the breast tumor tissue. Therefore, the applied algorithm deals better with large tumors that concentrate EM energy, but also offers a satisfactory solution in the case of smaller tumors. The performed simulations demonstrated that the breast tumor could not be heated evenly across its entire volume. The heterogeneity in tumor temperature distribution is mainly due to the different perfusion rates of tissues surrounding the tumor and, thus, different levels of cooling around the peripheral tumor layer. This reiterates the fact that each breast cancer clinical case should be considered independently, and various factors including tumor size, shape, locations, and physiological aspects, as well as the physico-thermal properties of the tumor and the healthy surrounding tissue, should be taken into account, as these factors influence the focused heating of the tumor tissue. The multi-objective optimization procedure can be considered as a critical tool for prospective regional hyperthermia treatment planning, i.e., to stipulate the crucial parameters for the dipole antennas for the expected therapeutic effects inside female breast tumors before actual targeted MW hyperthermia treatment is initiated in clinics. 

## Figures and Tables

**Table 1:** Dielectric and thermal tissue parameters employed in a female breast phantom for dipole antennas working at a frequency *f* = 1 GHz [61].

Tissue	*ε*_r_	*σ* (S/m)	*ρ* (kg/m^3^)	*C* (J/kg/K)	*k* (W/m/K)	HTR * (mL/min/kg)	*ρ*_b_*C*_b_*ρ*HTR (W/m^3^/K)	HGR ** (W/kg)
Blood	61.06	1.583	1050	3617	0.517	10,000	6.646·10^5^	0
Breast fat	5.41	0.053	911	2348	0.209	47	2710	0.728
Breast gland	59.47	1.079	1041	2960	0.334	150	9884	2.323
Fat	11.29	0.116	911	2348	0.211	33	1903	0.507
Muscle	54.81	0.978	1090	3421	0.495	37	2553	0.906
Skin	40.94	0.900	1109	3391	0.372	106	7441	1.648
Tumor ***	54.81	0.978	1090	3421	0.495	see Equation (3)	see Equation (3)	0.906

* heat transfer rate, ** heat generation rate, *** tumor was modelled using the muscle tissue parameters.

**Table 2:** The optimized parameters of an eight-dipole antenna array for various breast tumor sizes.

	**Tumor Size** ***r*** ** = 2 mm**			**Tumor Size** ***r*** ** = 4 mm**	
**Antenna No.**	**Power** *P_i_* (W)	**Phase** *ϕ_i_* (°)	**Antenna No.**	**Power** *P_i_* (W)	**Phase** *ϕ_i_* (°)
1	0.3113	9.1212	1	0.3202	9.8917
2	2.8172	−55.0652	2	2.8224	−53.5573
3	3.1238	7.5346	3	3.1074	8.0062
4	0.7207	122.8282	4	0.6914	124.1266
5	0.0015	155.1581	5	0.0014	159.7430
6	0.0252	82.6938	6	0.0257	77.6330
7	0.3572	30.1722	7	0.3668	28.1556
8	0.6431	0	8	0.6647	0
	**Tumor Size** ***r*** ** = 6 mm**			**Tumor Size** ***r*** ** = 8 mm**	
**Antenna No.**	**Power** *P_i_* (W)	**Phase** *ϕ_i_* (°)	**Antenna No.**	**Power** *P_i_* (W)	**Phase** *ϕ_i_* (°)
1	0.3491	12.0320	1	0.3932	12.6731
2	2.8912	−51.2217	2	3.2524	−53.2903
3	2.9268	10.5009	3	2.6419	9.9976
4	0.6478	128.7051	4	0.5701	135.6660
5	0.0011	179.4337	5	0.0020	−147.9632
6	0.0243	70.5049	6	0.0105	61.6180
7	0.3954	25.5423	7	0.3104	26.5520
8	0.7643	0	8	0.8195	0
	**Tumor Size** ***r*** ** = 10 mm**			**Tumor Size** ***r*** ** = 12 mm**	
**Antenna No.**	**Power** *P_i_* (W)	**Phase** *ϕ_i_* (°)	**Antenna No.**	**Power** *P_i_* (W)	**Phase** *ϕ_i_* (°)
1	0.4054	14.6487	1	0.3320	23.0597
2	3.7182	−52.4283	2	4.3765	−43.1907
3	2.4654	8.4417	3	2.4697	11.1388
4	0.4516	140.2195	4	0.2764	148.3518
5	0.0036	−125.5640	5	0.0077	−99.0500
6	0.0066	12.1489	6	0.0456	−37.1462
7	0.1896	18.0303	7	0.0842	−42.1068
8	0.7596	0	8	0.4079	0

## Abbreviations

AC	alternating current
AJCC	American Joint Committee on Cancer
BRCA1	breast cancer type 1 susceptibility protein
BRCA2	breast cancer type 2 susceptibility protein
BVM	boundary volume method
CT	computed tomography
ED	extremely dense female breast
EM	electromagnetic
EMF	electromagnetic field
FDTD	finite-difference time-domain
FEM	finite element method
FOCO	Focusing via Constrained power Optimization
FVM	finite volume method
GLOGBCAN	global cancer database
HD	heterogeneously dense female breast
HDI	Human Development Index
HGR	heat generation rate
HTR	heat transfer rates
ICD	International Classification of Diseases
IT’IS	Information Technologies in Society
MNPs	magnetic nanoparticles
MRI	magnetic resonance imaging
MW	microwave
PEC	perfect electric conductor
PF	predominantly fatty female breast
PML	perfectly matched layer
PSO	swarm-based optimization
RF	radiofrequency
ROI	region of interest
SAR	specific absorption rate
SFG	scattered fibro-glandular female breast
TNM	tumor-nodes-metastasis
WHO	World Health Organization
2D	two-dimensional
3D	three-dimensional
